# Oral Care Experiences of Children with Down Syndrome: Caregiver and Dentist Perspectives

**DOI:** 10.3390/healthcare13090999

**Published:** 2025-04-26

**Authors:** Marinthea Richter, Elizabeth Isralowitz, José C. Polido, Sharon A. Cermak, Leah I. Stein Duker

**Affiliations:** 1 Chan Division of Occupational Science and Occupational Therapy, University of Southern California, Los Angeles, CA 90089, USA; 2 Division of Dentistry, Children’s Hospital Los Angeles, Los Angeles, CA 90027, USA

**Keywords:** Down syndrome, dental care, oral care, children with special healthcare needs

## Abstract

**Background/Objectives**: Children with Down syndrome (DS) have distinct oral care needs and challenges, yet research on their care experiences, exploring caregiver and provider perspectives, is limited. Therefore, this study aimed to describe the barriers and facilitators to oral care for children with DS, as reported by caregivers and dental professionals. **Methods**: In this qualitative inquiry, semi-structured questions were used to elicit narratives describing oral care experiences from one caregiver focus group (n = 5), individual caregiver interviews (n = 9), and individual dentist interviews (n = 8). The transcripts were coded and thematically analyzed. **Results**: Three themes emerged in both groups. The first theme, *Access,* described the challenges in locating a dentist willing and knowledgeable about how to treat children with DS, and the variability in experiences between different contexts (i.e., community-based vs. specialty clinics). The second theme, *Pre-visit Preparation*, noted the potential impact of dental trauma on dental visits and recommended the use of preparation strategies, such as desensitization appointments, strategic scheduling, and visual or verbal scripts or social stories, to introduce dental encounters. The final theme, *Dental Encounters*, dealt with the importance of communication and interpersonal connection, as well as concerns about and support for active/passive immobilization techniques and pharmacological intervention. Sensory strategies for auditory, tactile, and vestibular input were discussed, in addition to distraction techniques, the timing and pacing of dental encounters, and parental presence/absence. **Conclusions**: Tailoring dental care around the unique sensory and behavioral needs of children with DS and building effective partnerships between children, parents, and dentists were emphasized for optimizing the dental care experiences of children with DS.

## 1. Introduction

Despite increasing utilization of dental visits, children with special health care needs (CSHCNs) in the United States present with more unmet dental needs and an increased prevalence of poor oral health than their typically developing (TD) peers [1,2]. Within the United States, about one in every 643 births (approximately 5,713 infants per year) is born with Down syndrome [3]. Children with Trisomy 21, or Down syndrome (DS), may be especially susceptible to oral health care challenges [4]. Although children with DS are reported to experience lower rates of caries than their TD peers, specific characteristics related to DS (e.g., immunological and metabolic differences, anatomical variation related to the oral structures, obstructive sleep apnea) have been linked to higher occurrences of oral health conditions, including gingivitis and periodontal disease [4,5]. 

While early access to dental care and the maintenance of oral health for children with DS has been emphasized [4], parents have reported challenges in utilizing these services, including the cost of care, insurance, discontinuity of care, and behavioral reactions and anxiety exhibited at the dentist [6,7,8,9]. However, research exploring dentists’ perspectives on caring for this population is sparse [10]. 

Minimal previous research has described the barriers to oral health care for American children with DS through caregiver-reported surveys [8,11], but as of this time no qualitative studies have examined the experiences of caregivers and/or dentists as they relate to the oral health of children with DS. Inquiries related to this topic have been conducted in other countries [9,12,13,14]; however, a qualitative study of American experiences is called for as the contextual factors related to healthcare systems may differ significantly. In addition, there is a need to evaluate caregiver and dentist perspectives in tandem to elicit multiple viewpoints. Therefore, this qualitative study utilized a thematic analysis to describe the barriers to and facilitators of oral care for children with DS, as reported by caregivers and dental professionals, to highlight their lived experiences and provide helpful strategies to enhance oral care for children with DS. 

## 2. Materials and Methods

### 2.1. Participants and Sampling

This exploratory study aimed to answer a narrow research question with great specificity (i.e., oral care experiences of caregivers of children with DS and the dentists treating this population). Therefore, based on the information power model suggested by Malterud and colleagues [15], the sample size was pre-determined to be approximately 6–8 caregivers and dentists.

#### 2.1.1. Caregivers

Caregivers of children aged 2–18 years with DS, with at least one previous dental cleaning, living in the Los Angeles area, and fluent in English were recruited using convenience sampling. Potential participants were identified from multiple sources, including respondents from an online oral health survey about children with DS [8], patients of the dental clinic at a large urban children’s hospital, social media DS group posts, this study’s website, and a local special needs resource fair. Target recruitment was two focus groups of 6–8 participants. However, due to scheduling challenges, only one focus group was scheduled (n = 5 caregivers). Caregivers unable to be scheduled were offered individual interviews either in-person (n = 4) or virtually (n = 5). The focus group and in-person interviews were conducted at the University research site; virtual interviews were conducted via a videoconferencing platform (i.e., Zoom). 

#### 2.1.2. Dental Professionals

Licensed dental professionals (e.g., dentist, dental hygienist) who spoke English and had treated at least 10 children with DS in the last year were recruited through emails sent to members of the Down Syndrome Association of Los Angeles and local dental providers (i.e., colleagues of research team members, pediatric dentists identified through web searches). Target recruitment was eight individual interviews with dental professionals, which was achieved. The interviews were conducted at the University research site or at the participants’ dental offices.

### 2.2. Data Collection

Immediately prior to the focus group/interview, caregivers and dentists completed a survey developed for this and previous studies [16] by our research group to obtain demographic and descriptive characteristics of participants (e.g., caregiver/child sex, caregiver/dentist education, child age, child race, presence/absence of specialized dental residency; see Table 1). The semi-structured interview/focus group questions were minimally adapted from a study describing the oral care experiences of autistic children [16], changing only the diagnosis from autism to DS. Questions were reviewed by an expert pediatric dentist and a qualitative research expert. Questions were open-ended, focusing on challenging and successful previous experiences as well as strategies to support care (e.g., What are the strategies you use at the dentist’s office for your child with DS that are most successful and why? What do you feel is the hardest part of oral care at the dentist for children with DS and why?; see Supplementary Materials). Prompts were utilized as necessary to elicit additional information. The same semi-structured interview questions were used for both the focus group and interviews.

The caregiver focus group was 56 minutes and individual interviews were an average of 40 minutes (SD = 11.3). Dentist interviews lasted an average of 51 minutes (SD = 7.3). The focus group and all interviews were audio-recorded and transcribed verbatim.

### 2.3. Data Analysis

Thematic analysis [17] was used to describe the oral care experiences shared by caregivers and dental professionals. Two coders independently read three caregiver and three dentist transcripts to develop two separate preliminary codebooks, followed by iterative discussion and a subsequent team meeting with a co-investigator to refine code definitions and finalize the codebook. Any additional changes to the codebooks were discussed and documented. Two independent coders coded all transcripts using QSR International’s NVivo qualitative data analysis software (version12). After all transcripts were coded, queries were performed to highlight incongruencies, and discrepancies were discussed until a consensus was reached. Codes applied across the dentist interviews and caregiver data were reviewed and grouped into themes. To enhance the rigor of the analysis, negative case analysis, consensus-driven thematic development, and an audit trail were utilized in addition to independent co-coding [18].

## 3. Results

### 3.1. Participants

Fourteen caregivers and eight dentists were enrolled and completed all study activities. The caregivers were primarily female (79%). Over half of the children with DS were female, between 8 and 11 years old, White, not Hispanic/Latino, and communicated primarily using single words or phrases. The dentists were primarily pediatric dentists with a mean of 23 years (SD = 11.5) of experience who reported treating children with DS very often in their practice (63%). Most of the dentists had post-graduate training, including a specialized residency with children with special health care needs (see Table 1).

### 3.2. Thematic Analysis

Three themes depicting the oral care experiences of children with DS emerged in both participant groups (see Table 2). 

#### 3.2.1. Access

##### Finding “The Right Dentist”

Accessing dental care was a priority for all the participating caregivers. One caregiver explained that “…maybe the hardest [time] I went through was when it had to do with looking and finding a good dentist…”. Locating “the right” dentist was a prolonged process in most cases, with one caregiver describing the “nightmare” of having to trial “about 15 dentists” in order to finally find one to treat their child. Another caregiver stated that their child was turned away from a community-based dental office and was seen as a “liability…more than a normal child that doesn’t have a lot of health problems. They don’t like servicing us, it seems”. A third caregiver explained that

I would call offices that I would get from a referral list from the insurance company, and I would just ask them, “Are you comfortable working with a child with special needs?” And eight out of ten would say, no, we don’t have anyone trained, we don’t have the necessary tools… A lot of them, thank God, were honest enough and said no, we can’t do it.

While all the dentists in the sample had treated CSHCNs, many affirmed the access challenges described by the caregivers, with one participant noting that “…by the time patients usually come to me, it’s because everybody else has said no to them…I’m sort of the last resort”. Two dentist participants specifically discussed transition-related challenges in finding a new provider when children with DS age out of pediatric care, with one participant explaining that “some…don’t want to leave, so I end up with 22-year-olds… and I still see them”.

##### Dentist Knowledge and Expertise to “Take Care of Kids with Special Needs”

All caregivers desired dental care provided by professionals with additional knowledge and expertise, with one caregiver explaining that “[Dentists] expect her [my child] to be treated the same. However, there are differences. As a mom, I would really love people knowing the subject…they know exactly what could happen, that they have the experience”. Another caregiver echoed these sentiments, describing that “I wish that the dentists would be more prepared in dealing with all kids, not just, you know, typical kids”. Several caregivers reported that many pediatric dental practices recommended by friends or family seemed ill-equipped to handle their child’s specific needs. For example:

So we started with a regular kid’s dentist…And it was hard…They were trying. But they weren’t…ready for him. They seemed like they didn’t know how to, you know, take care of kids with special needs. [But] they were great with my other son.

Related to this desire to find a knowledgeable and experienced provider, seven of the participating families’ children had received care at a specialty dental clinic at a local children’s hospital. One family described being fearful of receiving care in a general dental office due to their child’s heart condition and preferred the specialty clinic at the children’s hospital, with another describing this setting as their “only choice”. Conversely, four families accessed dental treatment at community-based dental practices that they felt were well-equipped to cater to their child’s needs. One caregiver specifically sought community-based care after an adverse experience at a hospital-based clinic. However, the majority of the families receiving care at specialty clinics reported that their child only received dental cleanings once a year due to long waiting times, despite more frequent visits being recommended. In contrast, two of the children receiving community-based care were able to access three to four cleaning visits per year by utilizing a combination of public and private insurance funding. 

One dentist participant surmised that hesitancy among community-based dental practitioners to treat children with DS may relate to co-occurring medical conditions and a lack of DS-related knowledge and expertise. However, this dentist felt that with a thorough medical history and adequate consults from the specialists involved in the child’s care, children with DS are “… just like another patient in practice”. In contrast to the majority of the caregivers’ opinions, one caregiver similarly stated that “…I don’t think they [dentists] do anything different. I guess they treat every kid the same”.

#### 3.2.2. Pre-Visit Preparation

Caregivers and dentists emphasized the importance of pre-visit preparation and practice, which were especially important due to previous medical/dental trauma. The impact of previous healthcare encounters—and the subsequent importance of preparing for dental visits—was described by one caregiver, who stated that “…because we strapped her down…those traumatic experiences really set her up to just not wanting to go,” while a dentist explained that “If I look at their health history, then I can understand why they don’t want to be there [at the dental visit]!” 

##### Desensitization Procedures: “Three Visits Before I Start Treating Him” 

More than half of the caregiver participants mentioned desensitization appointments, with multiple families (n = 5) having engaged in informal desensitization visits by having their child with DS accompany other family members to dental visits to observe and become familiarized with the environment, while two other families engaged in a structured, systematic desensitization processes with their child:

…she [the dentist] told me, “We’re going to do three visits before I start treating him”. So the first visit wasn’t even inside or sitting on the chair. It was in her office. And they started like talking and playing…10 minutes. Next time she took a little bit more time without me in there. And then, the third time, she could do the X-ray and check. No cleaning, no procedures. Then we went for the cleaning. So, she spent three visits to build rapport before treating him. And now he goes like very happy, and he goes in there and they say, “Hi, friend,” and he sits in the chair.

This dentist did not bill the family for these additional desensitization visits, but the caregiver noted that she would have been willing to pay for the dentist’s time investment to facilitate her child’s success. Another described their desensitization process, which involved both the dental team and a behavioral therapist:

So we started working with her behavior therapist to practice playing dentist with her. You know, getting comfortable reclining in a recliner—mirrors, and stuff, put in her mouth… I called the dentist…She had us come before they normally open and have other kids in the dentist’s office. And we tried for half an hour. We got her in the chair… Did, puppets and “Here’s Dolly getting checked-up”. Okay, great—”now it’s your turn”. She sat in the chair, and the second it went back, she flipped out and jumped out. Done. So, we kept working. Behavior therapists—six months later, we went back. And what they said was “She was great and very cooperative for the first five minutes”.

Echoing the caregiver results, the dentist participants also highlighted desensitization procedures, scheduling, and other preparation techniques to lay the groundwork for dental visits. Desensitization visits to address dental fear, anxiety, and cooperation were endorsed by the majority of the dentist participants, although they enacted them differently. For example, one dentist participant shared information about a government grant-funded systematic desensitization project to coach CSHCNs through dental appointments in non-dental settings, to enable their eventual treatment in a regular dental environment. Other dentists described desensitization visits within traditional dental practices, with one participant stating that they allow caregivers to bring children to the office to walk through and collect a toy at any time without a formal appointment, explaining that

…they just bring in the kid, and I don’t do anything...sometimes I just let them sit next to me, or get in the room. A lot of times, the first visit is desensitization—I don’t even want to bring them in the room.

Despite the enthusiasm for desensitization procedures, dentists mentioned the challenge of appropriate billing for desensitization and longer visits, as well as limited parental resources and time, which also negatively impacts the feasibility of multiple visits. One dentist participant suggested that having other staff members manage desensitization visits may minimize the impact on already lengthy waiting lists for routine dental appointments.

##### Strategic Scheduling at “The Best Time for That Patient”

Another preparatory strategy focused on strategic appointment scheduling. Two caregivers noted the benefit of going early in the morning or directly after lunch, perceiving that these time slots ensured the shortest waiting time and the least exposure to potentially distressing sounds of other children receiving treatment. The dentist participants also noted that they purposefully schedule appointments when their offices are quieter in the mornings, on specific days of the week, or whenever the parent believes is “the best time for that patient”.

##### Visual and Verbal Preparation Strategies

The caregivers described additional strategies they employed or recommended to prepare children for dental visits, including visual strategies (e.g., books, pictures) and utilizing familiar phrases, with one explaining that

Well, before, I didn’t even tell him that he was going to a dentist. We just arrived there and he started shaking…Now, I tell him, “Hey, you have a dentist appointment. We’re going to see your friend, and your teeth are going to sing”.

However, while verbal preparation was perceived as a useful strategy for some families, multiple other caregivers mentioned that their child would not necessarily benefit from this method. The dentists similarly highlighted visual and verbal strategies that could help caregivers to prepare their child for dental appointments, suggesting dentistry-related YouTube videos, “virtual tours,” “social stories,” and carefully crafted verbal scripts that

…don’t preemptively generate ideas of fear and anxiety. “It’s going to be okay. It’s not going to hurt. If you’re good, I’ll do this for you”. But now, they just initiated that idea in a child that, “Oh, this is something that could be scary. It’s something that could be hard. Because they want to reward me if I do well”. Rather just tell the kid, “We’re just going to go to the dentist. And they’re going to look at your teeth. And they’re going to brush them the same way you brush them at home”.

#### 3.2.3. Dental Encounters

This theme encompasses strategies and techniques used or recommended within the clinic and during the appointment. The importance of communication, patience, kindness, and interpersonal connection through playfulness permeated the discussions related to effective techniques.

##### Developing Rapport to “Make Him Feel Comfortable”

The dentists and caregivers spoke extensively about the significance of rapport and the atmosphere in the dental clinic, starting in the waiting room and continuing through the entire dental encounter. One caregiver described the following:

Upon arrival, the staff is so amazing. They’re, like, maybe not even in regular scrubs… They’ll greet him by his name. They come down to his level and look at him straight, eye-to-eye. They immediately make him feel comfortable…So that’s the number one thing…they exude so much positive energy and love.

Similarly, a dentist participant explained the importance of how to initiate a visit:

…the worst place to meet the children is in a dental chair. So we always meet them in a conference room, nicely decorated, comfortable. We let them sit there for a while with Mom, and…we’ll play a video game...eventually, we’ll walk into the clinical area…gradual step-by-step. We have…a hopscotch into the clinical area, it’s just kid-friendly.

Other dentist participants also emphasized the importance of meeting the child without wearing masks, loupes, or glasses; “trying to not act like a doctor”; building rapport; and tailoring communication to the child by “treat[ing] them like they understand, because they do understand. But simplify[ing] things so that they don’t feel intimidated”. The importance of this communication style was supported by the caregivers, with one stating that

It’s not a talk with the mom. Yes, [the dentist] instructs me with the specific medical or dental needs, but in order to do a cleaning, [the dentist] addresses the child and explains to the child what is going to happen. Not me, because I’m not in the chair, right?

One dentist also emphasized the importance of all team members—not just the dentist—employing these strategies in order to ensure the best possible outcome, explaining that

So basically I think it’s that friendly smile, and I always try to get the dental assistant that works with me to be friendly and accommodating as well because that’s part of the key. If you don’t have a good team, it doesn’t work as well.

Another caregiver described that having longer appointments to provide sufficient time to prioritize developing the child-dentist connection would be ideal, and that success was not due to any specific strategy (e.g., music, videos)—“None of that. It’s just the rapport. That’s what makes it different [successful]”.

##### Flexibility for “Adapted Seating Options”

Multiple dentist participants noted that care in a dental office does not necessarily translate to treatment in the dental chair. Considering the child’s positional preference was paramount, resulting in “a lot of stand-up dentistry” or exams occurring on the clinic floor, a regular chair, or a caregiver’s lap. The caregivers affirmed the need for adapted seating options, such as sitting or reclining with a caregiver or being positioned in a knee-to-knee position, with one caregiver even bringing a reclining chair from home for their child’s treatment. 

Half of the caregiver participants recounted experiences with active and/or passive immobilization (i.e., restraint by caregiver/staff vs. a stabilization device, respectively), voicing concerns about both. The caregivers described apprehension about the sustainability and safety of active immobilization given their child’s growth and strength, with one stating, “So I have to sit on the chair, put him on my lap, and grab him…which is very uncomfortable for me, for him, and for the dentist”. Although some of the caregivers felt uneasy about active immobilization techniques, those who had experienced passive immobilization often described these as causing adverse psychological effects, both for the child and caregiver:

Like she [the child] may not holler or scream out, but she’s showing it…the most traumatizing is when they strap her in. I don’t even stay in there. I just leave. I can’t see my daughter in pain. It’s too traumatic, not only for her but for me.

However, one caregiver recounted a positive experience with passive immobilization for her daughter:

They [dentist] tell me, well, we’re going to tie her [child] down just in case. And they did do that, they did tie her down. I told her that they were going to tie her so she wouldn’t move. That it was for her, to be comfortable. So, she understood, and she was willing.

Two dentist participants agreed that passive immobilization may be “harsh” due to a “loss of control” for the child and may lead to “dental phobia,” reporting that they avoided using passive immobilization “most of the time”. However, another dentist mentioned the potential benefits of passive immobilization, describing that the deep pressure sensations could calm some patients, describing a positive experience for an 18-year-old patient when “…she walked into the room, laid down on the papoose board, and waited for Mom and I to wrap her up. Both caregivers were almost in [happy] tears. I mean, it was really so comfortable and so easy”.

##### Sensory Strategies: “Sound Is Always a Problem”

The caregiver and dentist participants also highlighted the challenges children with DS face when encountering sensory stimuli in dental offices and discussed strategies to promote positive experiences. To address auditory sensitivities, one dentist described using tell-show-do to introduce equipment that may be noxious:

Sound is always a problem because everything’s air-driven. Even if you get an electric drill, it’s still going to whistle…I said “Okay, so, this is the one that dances on your tooth, you know, polishes and cleans”. And I’ll put it by their ear, and I’ll run it. I’ll put their hand around and…I said “It dances a little bit in your hand, doesn’t it?”

Both the caregivers and dentists were cognizant of heightened oral tactile sensitivity, explaining that “just having anything in the oral region can be highly sensory stimulating”. This included the traditional dental equipment required to complete care, dental-related equipment required for safety (e.g., “a bite block in the mouth is added sensory involvement”), and the dentist’s fingers, with one caregiver describing that “when [her child] has somebody maneuvering, putting their hands in her mouth…that’s the hardest”. To alleviate some of this discomfort, multiple dentists suggested letting the children hold the equipment (i.e., the mirror) or “hold onto my hand as I’m doing a prophy or a cleaning just because it gives them a little bit more control”. 

Vestibular (movement)-related challenges were also identified by the dentists, with one describing “…that motion going backward definitely brings a barrier to them. And so we’ll often have them sit at a 45-degree angle [instead of a full recline]”. In addition, the benefits of calming visual stimuli were mentioned by a caregiver who recounted that the dentist “took the lights from white…to maybe an orange or blue or something, and it was so very, very soothing and calm”.

##### Distraction: “Find That YouTube Video They Like”

Utilizing distraction techniques, specifically music, videos, or comfort items from home, was endorsed by both the caregiver and dentist participants, with one dentist noting that “we always encourage patients to bring music, or if they have a blanket they’d like to have”. The use of technology for distraction was also seen as beneficial for both the child and the dentist, with one caregiver lauding their dentist’s office because they “…interview each family and find out what motivates the kid…my son will either listen to Lion King, football…and they put that on every screen”. This was echoed by a dentist participant who recommended providers “find that YouTube video they like—that’s your distraction. You don’t have to work on saying nonsense sentences when you’re trying to concentrate…You just let the movie or song do it for you”. In addition to the use of technology, two caregivers emphasized using imaginative play as a distraction technique. Unfortunately, other caregivers noted that they did not have the chance to try distraction techniques, with one explaining that “…honestly, there’s no time. He’s already kicking and fighting. And we just hold him [so] he won’t get hurt”.

##### Timing and Pacing: “Tolerance Time”

The dentist participants frequently noted the importance of controlling the duration and pacing of dental visits. For example, the dentists highlighted counting as a strategy to give “a time frame of how long we’re going to be doing this so that they know, okay, five more seconds”. Children’s behavioral cues also helped dentists determine when to pause or end a visit:

We also call it the “tolerance time”. And for some kids, you [can] go for five minutes, and it’s no big deal. Others, you find, that if you stop at this particular point—give them time to take a breath and relax a little bit, get sort of reorganized, then you start again.

One dentist echoed this “tolerance time” sentiment, emphasizing that shorter, more frequent visits may support success:

…they can’t sit through long appointments…they’re communicating…“I’ve had it for today”…so okay, let’s put on fluoride varnish or do whatever we need to do and “We’ll see you next time”…you don’t want to force them…that’s not going to be successful. You want them to have pleasant appointments.

While the caregivers appreciated when dentists allowed breaks during their child’s appointment, several caregivers voiced disappointment that visits were shortened without allowing adequate time for their child to acclimatize to the environment or process verbal instructions:

She [dentist] wanted [my child] to open her mouth right now…I know that [my child] needs five seconds—then [the dentist] said, “Okay. That’s it. No, I’m sorry, Mommy and Daddy, we can’t do it”. Then, at that time, when [the dentist] was leaving, [my child] was like [gestures: opens mouth].

##### Parental Presence and Absence: “I’m Treating the Parent as Well”

Most of the dentist participants viewed parental presence as comforting and reassuring and noted that caregivers are often helpful for behavior management and active stabilization techniques when needed. One dentist emphasized that a parent’s presence is vital, stating that “I’m not only treating the child, I’m treating the parent as well. So, I have to make sure that they have confidence in what I do and what I don’t do”. Similarly, the majority of the caregivers valued being in the room during dental treatments to support their child’s comfort and safety, with most describing that their role during dental care was to help their child regulate and cooperate.

However, the dentist participants cautioned that caregiver anxiety, threats from caregivers to encourage compliance (e.g., “if you don’t behave, she’s [the dentist] giving you a shot”), and excessive coaching could disrupt the dental visit and challenge the rapport they had built with the child. One dentist endorsed the occasional use of parental absence, while others noted that they sometimes asked caregivers to be silent observers, explaining that multiple people talking could overwhelm the child. Three caregivers also discussed parental absence. One caregiver relied on their child’s behavioral therapist to accompany their child in an effort to minimize the number of people in the dental office. The other two caregivers emphasized their child’s need to develop confidence and independence in healthcare settings, explaining that “I’m hoping that eventually I can stay outside and he can go in by himself to the [dental] cleaning…That hasn’t happened yet, but I’m just hoping…I need him to be independent and trust his physicians”. 

##### Pharmacological Techniques: “Take Me into Consideration on the Decision”

For six children in this study, oral care involved general anesthesia in an operating room. Two families recounted positive experiences, with one caregiver stating that their daughter woke up without pain or adverse effects, explaining that “…if she would have to be tied down or get it done with her being alert, I think it would’ve…maybe traumatized her…she wouldn’t be able to be taken to the dentist as smoothly”. Similarly, another caregiver explained that “For us, it was the easiest thing, we could put him to sleep…he didn’t go through…being traumatized…he was asleep, he woke up, he was in a lot of pain, but it was better”. 

However, several caregivers reported a loss of agency and felt the decision to utilize pharmacological methods took place without their input, with one caregiver describing that “They [the dentist] just said, “…She’s not going to open her mouth. Sedate her,” without trying to complete care without drugs. Other caregivers described the use of pharmacological methods as “scary,” with one recounting her daughter’s experience after general anesthesia, stating that “I bring her home. And she went to try to stand up and she [couldn’t]. And I’m like this isn’t good, this shouldn’t be dentistry”. In addition, both a lack of communication and “the cost and charges of the procedure” increased caregiver stress, with one caregiver explaining that “We didn’t know until they sedated her. They found out that she needs all this. So they did it when she was under. So they did not tell us anything”. Another caregiver recounted a negative experience where communication between the dental team in the operating room and the caregiver was suboptimal, leading to an erosion of trust:

…they said that they will have the anesthesia to try to repair the teeth or remove the ones that aren’t like very well…They didn’t even ask me anything and they extracted all, all the upper teeth. All of them! It was just like, what the hell?

That same caregiver advocated for improved communication and caregiver involvement in decision-making processes, stating “Go out and say, “You know, these are the findings, Mom. Can we remove all the teeth because these are the pros and the cons?” Take me into consideration on the decision”.

Interestingly, several dentist participants commented not on the use of pharmacological techniques, but rather the efforts and strategies employed to minimize the need for pharmacological techniques, with one dentist explaining that

We really are fortunate because we invest a lot of time and resources into trying to stay out of the operating room under general anesthesia. And I consider it a success if we’ve gotten to that point with that patient that we can do that cleaning in the office, and their examination.

One strategy frequently discussed was silver diamine fluoride (SDF), with one dentist explaining that “We can SDF it multiple times. Get them back for a cleaning. Just check to make sure it’s hard or not, and then if it’s hard, then you save them an OR [operating room] visit”. Another strategy was related to the previously discussed theme of timing and pacing, with a dentist describing multiple shorter appointments for preventive care to reduce the use of pharmacological methods, explaining that

So, if we’re doing something that’s non-invasive, such as sealants for their teeth, maybe I’ll do, you know, I’ll even do just one and have them come back and just tell the parent that we’re going to do four visits, one tooth at a time, and it’s just going to be easier for them and we’re going to be able to do something preventative for them so that, you know, we can avoid going to the operating room or whatever is necessary.

However, the dentist participants shared that, in some cases, when other options have been exhausted and more invasive treatment is required, pharmacological techniques are often necessary. For example, one dentist stated that “…coming to extractions, restorations, and all that? That’s why God invented anesthesiologists. We go there and have them do that,” while another specified that “…success is being able to see them every six months like any other child, and to do a good cleaning, good examination, and when, obviously, there’s things to be done, they go off to the OR”.

## 4. Discussion

This study juxtaposes the experiences reported by caregivers of children with DS and dentists treating this population. Access, pre-visit preparation, basic behavioral techniques implemented in waiting rooms and dental offices, and advanced behavioral guidance strategies implemented during dental treatments were discussed by both groups, highlighting both negative and positive experiences while emphasizing individualized care and approaches that have been successful.

Within the United States, barriers, such as the cost of care, insurance-related issues, and difficulty locating dental professionals who are willing to treat and are knowledgeable about children with DS have been identified [7,8]. Echoing these previous reports, the caregivers in this study also shared their difficulties in finding a dentist who was both willing and able to treat their child. While some reported accessing community-based services, most relied on specialty hospital-based options, with varying opinions on the most suitable dental care setting for this population. These mixed views on the ideal dental care setting are consistent with those reported by caregivers of adults with DS [13]; however, they contrast with views expressed by Belgian parents of children with DS, only 10% of whom felt that specialized dental care was indicated [6]. 

Previous research has highlighted that the anxiety and behavioral reactions of children with DS at the dentist complicate care [8,9]. Most of the caregiver and dentist participants supported the use of preparatory techniques, including desensitization, the provision of information about dental visits (e.g., social stories, books, videos), and/or direct observation/modeling. These results are similar to a recent survey of pediatric dentists endorsing the routine use of these strategies (75%, 63%, and 80%, respectively), with rare instances of hesitancy, reluctance, or refusal by the caregivers [19]. Acclimatization visits have been recommended for dental practitioners treating children with DS [20], and the caregivers and dentists in this study described both informal as well as individualized, systematic desensitization processes. Other studies have also reported on practice dental visits for children with DS, though more research on the efficacy and optimal format, content, structure, timing, and frequency of the interventions specifically for this population may be indicated [7,9]. In contrast to the desensitization practices categorized as requiring “low resource utilization” in the American Academy of Pediatric Dentistry’s (AAPD’s) practice guideline recommendations [21], their cost, parent resources, and extended time demands were identified as potential barriers by our participants. As the American Dental Association creates new codes for behavior, desensitization, and education-related techniques in dental offices, it is hopeful that insurance companies may begin to reimburse for these activities. In addition to practice visits, some of the dentists and caregivers in our study listed verbal preparation and social stories as feasible techniques independent of desensitization visits. Social stories have been reported to positively impact oral health-related behaviors for preschool CSHCNs [22,23] and have been recommended for dental practitioners treating children with DS [20]. Despite the enthusiasm for these techniques voiced by our participants and echoed in other literature, the AAPD’s clinical practice guideline recommendations on nonpharmacological behavior management [21] reports only small effects of desensitization techniques on the behavior and/or anxiety of CSHCNs, and no specific recommendation is given regarding the pre-visit provision of information due to a lack of evidence. However, the AAPD’s recommendation does note that clinical reasoning, patient factors, and parental choice should guide the implementation of these techniques [21].

Other basic behavior guidance techniques described in our study include verbal and non-verbal communication, tell-show-do, positive reinforcement, distraction, sensory-adapted dental environments, and parental presence/absence. While the topic of communication permeated the discussions, the caregivers emphasized directing communication to the child, simplifying language, acting playfully, and allowing additional time for children to respond to instructions. These findings align with other reports noting that sympathy, reassurance, rapport, and inclusivity are valuable interpersonal strategies to use when treating people with DS [6,9,13]. Distraction coupled with technology and basic sensory-based environmental adaptations were supported by the caregivers and dentists. The AAPD recommends the use of these techniques for CSHCNs, despite noting the low certainty of the evidence and a need for further research on most of the strategies [21]. Parental absence as a behavior management technique was met with hesitancy, with most of the dentists and parents preferring to have parental presence for the safety, security, and comfort of the child. Another study has supported the importance of parental presence, specifically for children with DS, noting that parents know their child best and can assist dentists in interpreting their child’s verbal and nonverbal communication [9]. Two parents from the current study noted a desire for parental absence, not as a behavior technique but rather as part of the scaffolding for their child’s future autonomy at dental appointments. 

Beyond the basic behavior techniques, the caregivers and dentists highlighted pacing and scheduling as additional strategies to increase cooperation. Having extended time available for visits was described as imperative by both the caregivers and dentists, but not without potential financial ramifications. Spending as little time as possible in the waiting room before treatment and careful consideration of the child’s tolerance time in the dental chair were salient points. The dentists in this study tended to favor ending a visit if the child was uncooperative in an attempt to limit or avoid potential trauma. In contrast, the caregivers in the current sample and a previous study expressed dismay at dental visits ending prematurely [9]. Despite a recommended frequency of at least bi-annual preventive dental visits [24], most of the children in our sample had only one dental appointment per year. These results also differ from other findings reporting that about 50% of children with DS have at least two dental appointments per year [7].

Advanced behavior guidance techniques, such as active and passive stabilization and the use of general anesthesia, were discussed in the current study. While some positive anecdotes related to passive stabilization and dental care under general anesthesia were mentioned, the majority of the parents spoke with apprehension about their family’s experiences with these advanced behavior guidance techniques. This uneasiness is in line with other parental reports describing mixed reactions to the use of passive stabilization for TD children and CSHCNs [25]. A small subset of dentists in this sample used passive stabilization in consultation with parents when other less restrictive strategies had been exhausted. This aligns with the recommendations by the AAPD, which describes passive stabilization as feasible but with a high probability of parental reluctance or refusal [21]. Several parents noted this technique was “traumatizing” for them and their children. Accordingly, dental visits have been identified in other studies as a potential source of family tension, with the parents of children with DS reporting guilt and distress related to their child’s oral health status, affecting their family’s quality of life [12,14].

In summary, our findings recommend the utilization of preparatory strategies (e.g., desensitization, provision of information about dental visits, observation/modeling) as well as additional techniques throughout dental treatments. During clinical encounters, several basic behavior guidance techniques were encouraged, including communication, distraction, tell-show-do, and environmental adaptations. Parental presence during dental encounters was strongly preferred, though parental absence was perceived as a potential method to prepare individuals with DS for future independence. Advanced behavior techniques were primarily met with apprehension, though positive outcomes were reported in select instances. Tailoring dental care around the unique sensory and behavioral needs of children with DS and building effective partnerships between children, parents, and dentists were emphasized to optimize dental care experiences for children with DS. 

This qualitative study provides valuable and nuanced details about the individual experiences of families with and dentists treating children with DS; however, limitations exist. For example, although the experiences of the participating families and dentists shed light on the barriers to and facilitators of positive dental encounters, suggesting possible strategies and areas for further research, the findings from this study cannot be generalized to the broader population of people with DS. Several families received care at the same children’s hospital, which may have biased the results. Only dentists with experience treating CSHCNs were recruited; therefore, the perspective of dentists who do not treat this population is not represented. The parents and children in the sample were not patients of the dentists interviewed; future research should recruit family-provider dyads to obtain differing perspectives of the same encounters. The caregivers and dentists were not interviewed together; although this allowed for an open sharing of experiences without potential challenges arising from dentist-patient power dynamics, conducting focus groups with both caregivers and dentists may allow for deeper reflection and more direct conversation. Lastly, future research should also include children’s perspectives.

## 5. Conclusions

This study highlights caregiver and dentist experiences related to the oral care of children with DS. Access challenges are discussed, along with the strategies used before and during dental visits to facilitate successful dental care for children with DS. Not surprisingly, the themes focusing on dental encounters echoed the AAPD’s behavior guidance best practices [26], adding nuanced recommendations regarding the successful and unsuccessful utilization of these techniques to the literature.

## Figures and Tables

**Table 1:** Participant demographic and descriptive characteristics.

Demographic and Descriptive Characteristics		N (%)
**Caregivers (N = 14) ^1^**		
**Sex**		
	Female	11 (79)
	Male	3 (21)
**Maternal education level (N = 11) ^1^**		
	High school or GED	4 (36.4)
	College	4 (36.4)
	Graduate degree or above	3 (27.3)
**Paternal education level (N = 11) ^1^**		
	High school or GED	6 (54.5)
	College	3 (27.3)
	Graduate degree or above	1 (9.1)
	Not reported	1 (9.1)
**Children (N = 11)**		
**Sex**		
	Female	7 (63.6)
	Male	4 (36.4)
**Age (years)**		
	5.0–7.11	1 (9.1)
	8.0–10.11	6 (54.5)
	11.0–13.11	4 (36.4)
**Child’s race**		
	White, Caucasian	6 (54.5)
	Asian	3 (27.3)
	Black, African American	2 (18.2)
**Child’s Hispanic status**		
	Not Hispanic/Latino	6 (54.5)
	Hispanic/Latino	5 (45.5)
**Child’s functional communication level**		
	Unable to communicate needs or wants	2 (18.2)
	Single words or phrases via vocalization, sign language, or AAC **^2^**	6 (54.5)
	Spoken sentences	3 (27.3)
**Dentists (N = 8)**		
**Years in practice** [mean (SD)]		23 (±11.5)
**Approximate number of children with Down syndrome treated in the last two years** [mean (SD)/median (range)]		149 (±155.3)/87.5 (10–500)
**Dentist specialization**		
	General dentist	3 (38)
	Pediatric dentist	5 (63)
**Post-graduate training ^3^**		
	Pediatric	4 (44)
	General	3 (33)
	Craniofacial	1 (11)
	None	1 (11)
**Specialized residency with children with special healthcare needs**		
	Yes	6 (75)
	No	2 (25)
**Did your education prepare you to work with children with special healthcare needs and children with Down syndrome?**		
	Yes	7 (88)
	No	1 (12)
**How frequently do you treat children with Down syndrome?**		
	Rarely	1 (13)
	Occasionally	0 (0)
	Often	2 (25)
	Very often	5 (63)

^1^ Total, n = 14 caregivers. However, three children were represented by two caregivers, with only one participating caregiver completing caregiver demographic questions. ^2^ AAC: augmentative and alternative communication. ^3^ One dentist listed training in both general and pediatric dentistry.

**Table 2:** Endorsement of themes stratified by participant group.

Themes		Caregivers ^1^ N (%)	Dentists ^2^ N (%)
**Access**			
	Finding the Right Dentist	11 (100.0)	5 (62.5)
	Dentist Knowledge and Expertise	11 (100.0)	6 (75.0)
**Pre-Visit Preparation**			
	Desensitization Procedures	7 (63.6)	7 (87.5)
	Strategic Scheduling	5 (45.5)	7 (87.5)
	Visual and Verbal Preparation Strategies	8 (72.7)	7 (87.5)
**Dental Encounters**			
	Developing Rapport	10 (90.9)	8 (100.0)
	Seating	9 (81.8)	8 (100.0)
	Sensory Strategies	9 (81.8)	7 (87.5)
	Distraction	10 (90.9)	8 (100.0)
	Timing and Pacing	8 (72.7)	8 (100.0)
	Parental Presence and Absence	10 (90.9)	8 (100.0)
	Pharmacological Techniques	9 (81.8)	8 (100.0)

^1^ Three children were represented by two caregivers; these caregiver dyads are represented as only *one* caregiver for the purpose of reporting the endorsement of themes. Therefore, the total N = 11 caregivers. ^2^ Total N = 8 dentists.

## Data Availability

The datasets presented in this article are not readily available because our study analyzed qualitative data which contain potentially identifiable information and the participants did not consent to having their full transcripts made publicly available. Excerpts of the transcripts relevant to this study are available to those who contact us with this request. Requests to access the dataset should be directed to lstein@chan.usc.edu.

## Supplementary Materials

The following supporting information can be downloaded at: https://www.mdpi.com/article/10.3390/healthcare13090999/s1, File S1: Sample Focus Group/Interview Questions.

## Abbreviations

The following abbreviations are used in this manuscript:

DS	Down syndrome
CSHCNs	Children with special health care needs
TD	Typically developing
AAPD	American Academy of Pediatric Dentistry
OR	Operating room
AAC	Augmentative and Alternative Communication
SDF	Silver diamine fluoride

## References

[1] Obeidat R., Noureldin A., Bitouni A., Abdellatif H., Lewis-Miranda S., Liu S., Badner V., Timothé P. (2022). Oral health needs of U.S. children with developmental disorders: A population-based study. BMC Public Health.

[2] Waldman H.B., Perlman S.P., Wong A. (2017). The largest minority population in the U.S. without adequate dental care. Spec. Care Dent..

[3] Stallings E.B., Isenburg J.L., Rutkowski R.E., Kirby R.S., Nembhard W.N., Sandidge T., Villavicencio S., Nguyen H.H., McMahon D.M., Nestoridi E. (2024). National population-based estimates for major birth defects, 2016–2020. Birth Defects Res..

[4] Botero J.E., Rodríguez-Medina C., Amaya-Sanchez S., Salazar C.L., Contreras A. (2024). A comprehensive review of the relationship between oral health and Down syndrome. Curr. Oral. Health Rep..

[5] Martins M., Mascarenhas P., Evangelista J.G., Barahona I., Tavares V. (2022). The incidence of dental caries in children with Down syndrome: A systematic review and meta-analysis. Dent. J..

[6] Descamps I., Marks L.A. (2015). Oral health in children with Down syndrome: Parents’ views on dental care in Flanders (Belgium). Eur. J. Paediatr. Dent..

[7] Hung M., Graves A., Lu J., Schwartz C., Lipsky M.S. (2025). Navigating barriers to dental care for patients with Down syndrome: A scoping review of challenges and strategies. Children.

[8] Stein Duker L.I., Richter M., Lane C.J., Polido J.C., Cermak S.A. (2020). Oral care experiences and challenges for children with Down syndrome: Reports from caregivers. Pediatr. Dent..

[9] Stensson M., Norderyd J., Van Riper M., Marks L., Björk M. (2022). Dental health care for children with Down syndrome: Parents’ description of their children’s needs in dental health care settings. Eur. J. Oral. Sci..

[10] Descamps I., Fernandez C., Van Cleynenbreugel D., Van Hoecke Y., Marks L. (2019). Dental care in children with Down syndrome: A questionnaire for Belgian dentists. Med. Oral. Patol. Oral. Cir. Bucal..

[11] Majstorovic M., Nandi S.S., Canares G., Chinn C., Szirovicza L., Best E., Moursi A.M. (2023). Oral health in the Down syndrome population: Parental perceptions on dental care in the United States. Pediatr. Dent..

[12] AlJameel A.H., Watt R.G., Tsakos G., Daly B. (2020). Down syndrome and oral health: Mothers’ perception on their children’s oral health and its impact. J. Patient. Rep. Outcomes.

[13] Kaye P.L., Fiske J., Bower E.J., Newton J.T., Fenlon M. (2005). Views and experiences of parents and siblings of adults with Down Syndrome regarding oral healthcare: A qualitative and quantitative study. Br. Dent. J..

[14] Oliveira A.C., Pordeus I.A., Luz C.L., Paive S.M. (2010). Mothers’ perceptions concerning oral health of children and adolescents with Down syndrome: A qualitative approach. Eur. J. Paediatr. Dent..

[15] Malterud K., Siersma V.D., Guassora A.D. (2016). Sample Size in Qualitative Interview Studies: Guided by Information Power. Qual. Health Res..

[16] Stein Duker L.I., Floríndez L.I., Como D.H., Tran C.F., Henwood B.F., Polido J.C., Cermak S.A. (2019). Strategies for success: A qualitative study of caregiver and dentist approaches to improving oral care for children with autism spectrum disorder. Pediatr. Dent..

[17] Braun V., Clarke V. (2006). Using thematic analysis in psychology. Qual. Res. Psychol..

[18] Padgett D.K. (2012). Qualitative and Mixed Methods in Public Health.

[19] Randall C.L., Dhar V. (2023). Pediatric dentists’ use of nonpharmacological behavior guidance techniques and experiences with parent/caregiver acceptance: A national survey. Pediatr. Dent..

[20] Brosnan S., Ray-Chaudhuri E. (2021). Parry, J. Dental care for children with Down syndrome: A guide for the dental practice team. Dent. Update.

[21] Dhar V., Gosnell E., Jayaraman J., Law C., Majstorović M., Marghalani A.A., Randall C.L., Townsend J., Wells M., Chen C.-Y. (2023). Nonpharmacological behavior guidance for the pediatric dental patient. Pediatr. Dent..

[22] Marion I.W., Nelson T.M., Sheller B., McKinney C.M., Scott J.M. (2016). Dental stories for children with autism. Spec. Care Dent..

[23] Zhou N., Wong H.M., McGrath C. (2020). Social story-based oral health promotion for preschool children with special healthcare needs: A 24-month randomized controlled trial. Community Dent. Oral. Epidemiol..

[24] American Academy of Pediatric Dentistry (2024). Periodicity of Examination, Preventive Dental Services, Anticipatory Guidance/Counseling, and Oral Treatment for Infants, Children, and Adolescents.

[25] Malik P., Ferraz dos Santos B., Girard F., Hovey R., Bedos C. (2022). Physical constraint in pediatric dentistry: The lived experience of parents. JDR Clin. Trans. Res..

[26] American Academy of Pediatric Dentistry (2024). Behavior guidance for the pediatric dental patient. The Reference Manual of Pediatric Dentistry.

