# Supplementary material for: Oral Care Experiences of Children with Down Syndrome: Caregiver and Dentist Perspectives
Source: Healthcare (Basel). 2025 Apr 26;13(9):999. doi: 10.3390/healthcare13090999 (PMC12072164; doi:10.3390/healthcare13090999)
Supplement: Supplementary file 1 [file healthcare-13-00999-s001.zip › healthcare-3582747-supplementary.pdf]

healthcare

MDPI

File S1: Sample Focus Group/Interview Questions

Sample Questions: Caregiver Focus Group/Interview

1. Tell me about your experience(s) finding a dentist.
2. Tell me about an experience with your child at the dentist that stands out in your mind.
3. What is the best part of the dental experience for your child and why?
4. What is hardest for your child at the dentist and why?
5. What are the strategies *you* use at the dentist's office that are most successful? Why do you think they are?
6. What are the strategies your *dentist* uses at the dentist's office that are most successful? Why do you think they are?
7. Does your child have any sensory difficulties? How do you think they affect his or her oral care?
8. If you have another child that does not have a diagnosis of Down syndrome, compare and contrast your dental office experiences with that child and your child with DS.

Sample Questions: Dentist Interview

1. Tell me how you came to be involved in providing care for children with DS.
2. Tell me about your experience(s) providing routine oral care to children with DS.
3. Tell me about a time when you were treating a child with DS that went the way you hoped it would/was successful. Tell me about a time that didn't go the way you hoped it would/was difficult.
4. What do you feel is the hardest part of oral care at the dentist *for children with DS*? Why?
5. What do you feel is the hardest part *for you* while performing oral care on children with DS? Why?
6. Approximately what percent of the time do you need to use anesthesia with children with DS in order to perform a routine oral cleaning? What is the primary reason?
7. Do you think that children with DS have more sensory sensitivities to bright lights, sounds, touch in and around the mouth, smells, taste compared to typical children you see? How do you know? How does this affect them and their care?
8. What are the strategies you use for children with DS that are most successful? Why do you think they are successful?
    a. Are there any strategies that you used with children with DS that *weren't* helpful/successful? Why do you think they weren't?
9. What are the strategies parents use at the dentist's office for their child with DS that are most helpful? Why do you think they are successful?
    a. Are there any strategies that parents used that weren't helpful? What were they and why do you think they didn't work?

 https://doi.org/10.3390/healthcare13090999
